# A potential protective effect of α-tocopherol on vascular complication in spinal cord reperfusion injury in rats

**DOI:** 10.1186/1423-0127-17-55

**Published:** 2010-07-07

**Authors:** Mohamed D Morsy, Ossama A Mostafa, Waleed N Hassan

**Affiliations:** 1 Physiology Department, College of Medicine, Menoufiya University, Egypt; 2 Public Health Department, College of Medicine, Beni Suef University, Eygpt; 3 Biochemistry Department, College of Medicine, Menoufiya University, Egypt

## Abstract

**Background:**

Paraplegia remains a potential complication of spinal cord ischemic reperfusion injury (IRI) in which oxidative stress induced cyclooxygenase activities may contribute to ischemic neuronal damage. Prolonged administration of vitamin E (α-TOL), as a potent biological antioxidant, may have a protective role in this oxidative inflammatory ischemic cascade to reduce the incidence of paraplegia. The present study was designed to evaluate the preventive value of α-TOL in IRI of spinal cord.

**Methods:**

For this study, 50 male Sprague-Dawley rats were used and divided into five experimental groups (n = 10): Control group (C); α-TOL control group (CE) which received intramuscular (i.m.) α-TOL injections (600 mg/kg); Sham operated group (S), IRI rats were subjected to laparotomy and clamping of the aorta just above the bifurcation for 45 min, then the clamp was released for 48 hrs for reperfusion; and IRIE rats group, received 600 mg/kg of α-TOL i.m. twice weekly for 6 weeks, followed by induction of IRI similar to the IRI group. At the end of the experimental protocol; motor, sensory and placing/stepping reflex evaluation was done. Plasma nitrite/nitrate (NOx) was measured. Then animals' spinal cord lumbar segments were harvested and homogenized for measurement of the levels of prostaglandin E_2 _(PGE_2_), malondialdehyde (MDA) and advanced oxidation products (AOPP), while superoxide dismutase (SOD) and catalase (CAT) activity were evaluated.

**Results:**

Induction of IRI in rats resulted in significant increases in plasma levels of nitrite/nitrate (p < 0.001) and spinal cord homogenate levels of PGE_2_, MDA, advanced oxidation protein products AOPP and SOD with significant reduction (p < 0.001) in CAT homogenate levels. Significant impairment of motor, sensory functions and placing/stepping reflex was observed with IRI induction in the spinal cord (p < 0.001). α-TOL administration in IRIE group significantly improved all the previously measured parameters compared with IRI group.

**Conclusions:**

α-TOL administration significantly prevents the damage caused by spinal cord IRI in rats with subsequent recovery of both motor and sensory functions. Alpha-tocopherol improves the oxidative stress level with subsequent reduction of the incidence of neurological deficits due to spinal cord IRI conditions.

## Background

Ischemic reperfusion injury (IRI) of the spinal cord occurs due to temporary interruption of the blood supply to the spinal cord. This may result in irreversible vascular injuries with subsequent paraplegia or other neurological deficits [1]. This serious complication is frequently seen in transient ischemic insults of the spinal cord and after surgical repair of thoraco-abdominal aortic aneurysms [2]. Oxidative stress with over-production of reactive oxygen species (ROS), such as free radicals and peroxides are incriminated in the neurological vascular injuries [3]. Increased ROS in dorsal horn neurons may contribute to central sensitization in neuropathic rats [4]. Overproduction of ROS and oxygen free radicals in ischemic reperfusion conditions may also lead to excessive lipid peroxidation and protein and DNA damage [5]. In rats, with ligation of sciatic nerve, superoxide dismutase (SOD) and glutathione peroxidase (GPx) activities increase, while catalase (CAT) activity decrease significantly due to associated oxidative stress and reduction of antioxidant defense potential [6]. In addition, Regan and Guo [7] reported that prolonged depletion of glutathione in the brain is associated with oxidative neuronal death. Ischemia induces oxidative stress, leading to induction and expression of various genes in a variety of cell types throughout the central nervous system [8]. One of these important genes is the cyclooxygenase enzyme gene. This enzyme is the rate-limiting enzyme involved in arachidonic acid metabolism, with subsequent generation of prostaglandins and thromboxanes that play important roles in sustaining the inflammatory response and induce different neurological deficits [9]. Elements of oxidative stress appeared to be essential for the activation of this enzyme [10]. Oxidative stress induces cyclooxygenase-2 (COX-2) activity in neurons after various CNS insults, including global ischemia [11].

The COX-2 inhibitors as SC-58125 and NS-398 have been shown to prevent delayed death of hippocampal neurons [12] and to reduce infarct size after global ischemia [13]. Vitamin E (α-tocopherol) is an important lipid-soluble chain-breaking antioxidant, essential to scavenge ROS in tissues, red cells, and plasma [14]. α-tocopherol (α-TOL) significantly reduces induced nitric oxide synthase enzyme (iNOS) activity and cyclic glutamate monophosphate (cGMP) levels in diabetic rats [15]. α-TOL, which is the predominant form of vitamin E in clinical uses, has been found to attenuate COX-2 activity by scavenging the oxidant hydroperoxide that acts as activator for COX-2 enzyme with subsequent suppression of prostaglandin E_2 _(PGE_2_) production [16]. Other experiments demonstrated that α-TOL decreases COX-2 activity through reduction of peroxynitrite formation necessary for its activation [17]. Another mechanism through which α-TOL reduces PGE_2 _production could be through direct inhibition of COX activity without affecting COX mRNA and its protein levels, indicating a post-translational regulation of COX [18].

Most of the carried studies in this field were constructed for short-term administration of the therapeutic agents just before or after IRI, while our study could be one of the recent studies for long-term prophylactic administration of α-TOL on IRI of the spinal cord.

So, the aim of the present study is to explore the possible prophylactic effect of long-term administration of α-TOL in spinal cord reperfusion injury against high oxidative stress induced by ischemia; and to investigate the role of α-TOL in the inhibition of COX-2 activity, with subsequent suppression of inflammatory PGE_2 _over-production.

## Methods

### Animals and their groups

This study followed a randomized controlled animal experiment design. A total of 50 male Sprague-Dawley rats were randomized into 5 study groups. All rats were obtained from the National Research Center, Cairo, Egypt and weighed between 150 and 200 gm. Animals were fed on a standard chow diet, water, *ad libitum *and housed in the animal house of Menoufiya College of Medicine with a 12:12-hrs light/dark cycle. The animals were randomly divided into five groups (n = 10 each) as follows: C rats which underwent no surgery; CE animals received α-TOL 600 mg/kg i.m. twice weekly [19]; S (sham) rats were subjected to laparotomy without clamping of the aorta; IRI rats were subjected to laparotomy and clamping the aorta by non-traumatic vascular clamp just above the bifurcation for 45 min, then the clamp was released for reperfusion for 48 hrs; and IRIE rats were injected i.m. with α-TOL by the same dose and maneuver as rats in the CE group then IRI was induced as in IRI group. Control rats were injected i.m. with vehicle alone. The experiments were conducted in accordance with the ethical guidelines for investigations in laboratory animals and were approved by the Ethical Committee of the College of Medicine, Menoufiya University.

### Induction of ischemic reperfusion injury of the spinal cord

Spinal cord ischemia was induced, as described by Akguna et al. [20]. Rats were initially anesthetized with i.m. ketamine (50 mg/kg), followed by a half dose as required during the procedure. The animals did not receive ventilatory support. Body temperature was monitored by a rectal probe inserted into the rectum and was maintained between 37°C and 38°C by a thermal pad and a heating lamp. The femoral artery was cannulated with a 22-gauge PE catheter, which was used to monitor distal arterial pressure (DAP) and for intra-arterial infusion of heparin. The left carotid artery was cannulated with a 20-gauge PE catheter (Terumo, Tokyo, Japan), which was used to monitor the proximal artery pressure (PAP) and to take blood samples. Each rat received 150 IU/kg heparin injected into the femoral artery immediately after completion of arterial cannulation and before aortic occlusion. The abdominal aorta was reached through midline laparotomy. Animals in sham group (group S) were anesthetized and subjected to laparotomy without aortic occlusion. In IRI and IRIE groups, animals were subjected to aortic cross clamping for 45 minutes. Vascular clamps were placed under the left renal vein and above the bifurcation of the aorta. The efficiency of occlusion was documented by an immediate and sustained decrease in DAP in the femoral artery. To maintain the PAP approximately at 40 mmHg during occlusion, blood from the carotid artery was allowed to flow into a collecting circuit filled with heparinized saline (4 U/ml of saline) positioned 54 cm above the rat. The temperature of the blood in the syringe was maintained at 37- 37.5°C. The aortic clamps were released after 45 min and the blood in the syringe was transfused back into the rat over a 60-sec period. After completion of all procedures, the wounds were closed. Protamine sulfate (4 mg) was subcutaneously injected to reverse the anticoagulation effect of heparin. Animals were allowed to recover in a plastic box at 28°C for 3 hours and were then placed in their cages with free access to food and water [21]. Rats with complete hind limb paralysis for 24 hours, hematuria, or 25% reduction in body weight were killed by using a lethal dose of thiopental sodium injection (75 mg/kg i.p.) for humanitarian reasons [22].

### Neurological assessment

Hind limbs neurologic function was assessed at 48 hours after the procedure using the Tarlov Scoring System [23]. A score of 0-5 was assigned to each animal as follows: 0 = no voluntary hind limb movement; 1 = movement of joints perceptible; 2 = active movement but unable to sit without assistance; 3 = able to sit but unable to hop; 4 = weak hop; 5 = complete recovery of hind limb function. The placing/stepping reflex (SPR) was assessed by dragging the dorsum of the hind paw along the edge of a surface. This normally evokes a coordinating lifting and placing response which was graded as follows: 0 = normal; 1 = weak; and 2 = no stepping [24]. Sensory function was assessed by a hind limb withdrawal from a stimulus (1 = withdrawal response to noxious stimulus applied to hind limbs and 0 = no response to noxious stimulus). One member of the research team who was blinded to the treatment groups conducted all neurological tests.

### Blood sampling and biochemical measurements

At the end of the experimental protocol period (48 hrs after IRI), retro-orbital blood samples were obtained through heparinized capillary tubes after overnight fasting. Samples were added to EDTA and were centrifuged at 1000 rpm for 15 min for separation of plasma and were stored at -80°C to assay total plasma nitrite/nitrate (NOx) level.

### Estimation of plasma Nitrite/Nitrate (NOx)

The method for estimation of total nitrite/nitrate (NOx) level was based on the Griess reaction. Plasma nitrite/nitrate levels were measured after enzymatic conversion of nitrate (NO_3^-^_) to nitrite (NO_2^-^_) by nitrate reductase in the presence of NADPH. The oxidation of the coenzyme was monitored by the decrease in absorbance at 540 nm. Results were expressed as μmol/L [25].

### Preparation of spinal cord homogenates

After completing the neurological assessment and obtaining the blood samples, all rats were killed using the lethal dose of thiopental sodium injection [22]. Sections of the 3, 4, 5 lumbar segments of the spinal cord were harvested, dissected out, cute into small pieces and homogenized using an Omni tissue homogenizer (Omni international, Gainesville, VA, USA) [26]. Tissues were homogenized in ice-cold lyses buffer [0.1 M phosphate, pH 7.4, 1 mM EDTA, 10 μM indomethacin (Cayman Chemical, Ann Arbor, MI, USA)] using a tube pestle. Acetone was added (2 × sample volume), and samples were centrifuged at 1500 × g for 10 min at 4°C. The supernatants were then stored at -80°C [27].

**Malondialdehyde (MDA),Advanced oxidation products (AOPP) levels, as well as SOD and CAT activities in Spinal cord homogenate**

Lipid peroxidation was assessed by the measurement of secondary product MDA after precipitation of protein by addition of trichloroacetic acid then thiobarbituric acid (TBA) which reacted with MDA to form TBA reactive product, which was measured at 532 nm. An MDA solution, freshly prepared by the hydrolysis of 1,1,3,3-tetramethoxy propane was used as a standard [28]. Determination of advanced AOPP (with characteristic absorbance at 340 nm) was based on spectrophotometric method detection (Shimadzu Corporation, Kyoto, Japan, UV- 160A) with tissue homogenate diluted with PBS to 0.2-0.5 g/ml [29]. SOD activity was assayed following the method of Kakkar et al. [30]. The sample containing 5 μg protein was mixed with sodium pyrophosphate buffer, phenazine methosulphate (PMT) and nitro blue tetrazolium (NBT). The reaction was started by the addition of NADH, incubated at 30°C and stopped by the addition of 1 ml of glacial acetic acid. The absorbance of the chromogen formed was measured at 560 nm. One unit of SOD activity is defined, as the enzyme concentration required to inhibit chromogen production by 50% in one minute per mg protein under the assay condition. CAT activity was measured in homogenate by the method of Bonaventura et al. [31]. 5 μg proteins from the homogenate was mixed with 2 ml of 7.5 mM H_2_O_2 _and a time scan was performed for 10 min at 240 nm at 25°C. One unit of CAT activity is defined as the amount of enzyme decomposing 1 μmol of H_2_O_2 _per minute per mg protein.

### Spinal cord homogenate PGE_2 _determination

Tissue levels of PGE_2 _in the spinal cord were assayed using monoclonal enzyme immunoassay (EIA) kit. The EIA kit demonstrates sensitivity from 10 to 1000 pg/ml and demonstrates little cross reactivity between structurally related PE_3 _and PE_1_. Absorbance (412 nm) values of standards and samples were corrected by subtraction of the background value to correct for absorbance caused by nonspecific binding [32].

### Chemicals

α-tocopherol was supplied by Sigma (St Louis, MO, USA) that utilizes polyoxyl-35- Ricinusolle as an emulsifier; ketamine hydrochloride was supplied by Pfizer Pharmaceutical Company, Inc, USA; thiopental sodium was supplied by Biocheme, Austria; heparin (Leo, Ballerup, Denmark); Protamine sulfate (Leo, Ballerup, Denmark); Prostaglandin E_2 _EIA kit and indomethacin (Cayman Chemical, Ann Arbor, MI, USA) and nitrate reductase from Aspergillus (Sigma).

### Statistical analysis

Data were expressed as frequency, percentage and mean ± SD. Testing significance was performed using χ^2 ^test and the one-way analysis of variance (ANOVA). Post-hoc Scheffe test was applied to identify the source of statistical significance. P-values < 0.05 were considered statistically significant.

## Results

**Figure 1:**
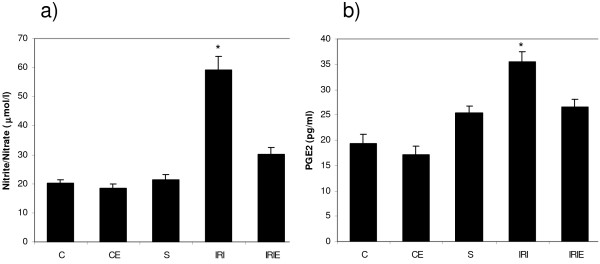
**Effect of α-Tocopherol treatment for 6 weeks in ischemic reperfusion injury in rats**. [a] Plasma level of nitrite/nitrate [b] Spinal cord homogenate levels of PGE_2; _. C: Control group; CE: α-Tocopherol control group; S: Sham group; IRI: Ischemic Reperfusion Injury group; IRIE: α-Tocopherol-treated ischemic reperfusion injury group. Results are expressed as mean ± SD (n = 10). The groups which carries the "*" is significantly different from all other groups (P < 0.001).

**Figure 2:**
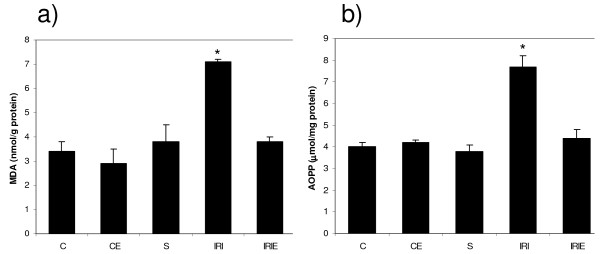
**Effect of α-Tocopherol treatment for 6 weeks in ischemic reperfusion injury in rats**. Spinal cord homogenate levels of [a] MDA [b] AOPP. C: Control group; CE: α-Tocopherol control group; S: Sham group; IRI: Ischemic Reperfusion Injury group; IRIE: α-Tocopherol-treated ischemic reperfusion injury group. Results are expressed as mean ± SD (n = 10). The groups which carries the "*" is significantly different from all other groups (P < 0.001).

**Figure 3:**
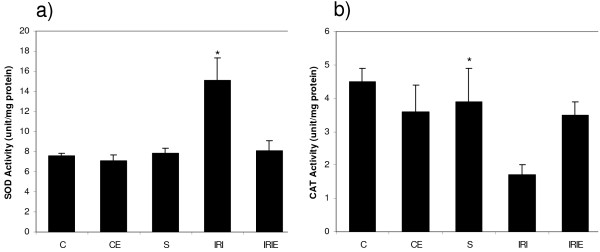
**Effect of α-Tocopherol treatment for 6 weeks in ischemic reperfusion injury in rats**. Spinal cord homogenate activity levels of [a] SOD; [b] CAT. C: Control group; CE: α-Tocopherol control group; S: Sham group; IRI: Ischemic Reperfusion Injury group; IRIE: α-Tocopherol-treated ischemic reperfusion injury group. Results are expressed as mean ± SD (n = 10). The groups which carries the "*" is significantly different from all other groups (P < 0.001).

In control and sham groups, the administration of α-TOL did not produce any significant changes in plasma nitrite/nitrate or spinal cord homogenate of MDA, AOPP and PGE_2 _levels, in addition to SOD activity and CAT activity. However, in sham operated group PGE_2 _increased significantly compared with the control groups (C, CE) (Fig. 1, 2, 3).

### Plasma nitrite/nitrate level

Induction of IRI in rats produced significant elevation of plasma nitrite/nitrate level (59.3 ± 4.5) compared with the control groups [C (20 ± 1.3), CE (18.4 ± 1.4) and S (21.5 ± 1.5), (P < 0.001)]. On the other hand, i.m. administration of α-TOL for 6 weeks resulted in significant reduction of the plasma nitrite/nitrate level compared with IRI group (33.2 ± 2.2) or control groups (P < 0.001) (Fig. 1).

### Spinal cord homogenate levels of MDA, AOPP and PGE_2_

Lipid oxidative product MDA (7.1 ± 0.1), protein oxidative product AOPP (7.7 ± 0.5) and inflammatory product PGE_2 _(35.4 ± 2.1) levels in spinal cord homogenate increased significantly in IRI group compared with the control groups [C (3.4 ± 0.4), CE (4.0 ± 0.2) and S (19.3 ± 1.8), (P < 0.001)]. While administration of α-TOL produced significant reduction of the previously mentioned parameters compared to the IRI group [(4.3 ± 0.4), (3.8 ± 0.2), (26.6 ± 1.4), p < 0.001] (Fig. 1, 2).

### Spinal cord homogenate activity of SOD and CAT

Induction of IRI in rats resulted in significant elevation of the SOD activity (15.1 ± 2.2) and significant reduction of CAT activity (1.7 ± 0.3) in the spinal cord homogenate compared with the control group [(7.6 ± 0.2) and (4.5 ± 0.8), respectively, p < 0.001 for both comparisons]. While, i.m. administration of α-TOL produced a significant reduction in SOD (8.1 ± 1.0) and a significant increase in CAT (3.5 ± 0.4) activities compared with the IRI group (P < 0.001 for both) (Fig. 1, 2).

### Motor, SPR and sensory assessment

In the present study, rats in the IRI group showed acute flaccid paraplegia of the hind limbs up to 6 hours after reperfusion injury followed by spastic paraplegia. Their motor score (mean ± SD) was 1.2 ± 1.0 compared with 4.8 ± 0.4 in the control group. Also, sensory function was impaired in 80% of rats in the IRI group, while SPR was lost in 60% and was impaired in 20% of IRI rats. On the other hand, α-TOL administration in IRIE group produced recovery of motor and sensory functions (3.8 ± 1.0 and 70%, respectively), while the SPR was recovered in 80% of IRIE rats (Table 1).

**Table 1:** Effect of α-Tocopherol treatment in ischemic reperfusion injury model in rats for 6 weeks on Motor function, Sensory response and Placing/Stepping reflex scores

	Test	C	CE	S	IRI	IRIE	p-value
**Motor assessment**	Mean ± SD	4.8 ± 0.4^(a)^	4.8 ± 0.4^(a)^	4.0 ± 1.1^(a)^	1.2 ± 1.0^(b)^	3.8 ± 1.0^(a)^	<0.001

**Sensory assessment**	Normal	8	9	7	2	7	
		(80%)^(a)^	(90%)^(a)^	(70%)^(a)^	(20%)^(b)^	(70%)^(a)^	
	
	Affected	2	1	3	8	3	<0.05
		(20%)^(a)^	(10%)^(a)^	(30%)^(a)^	(80%)^(b)^	(30%)^(a)^	

**Placing/Stepping reflex**	Normal	8	9	7	2	8	
		(80%)^(a)^	(90%)^(a)^	(70%)^(a)^	(20%)^(b)^	(80%)^(a)^	
	
	Impaired	2	1	3	2	1	<0.01
		(20%)^(a)^	(10%)^(a)^	(30%)^(a)^	(20%)^(a)^	(10%)^(a)^	
	
	Lost	0	0	0	6	1	
		(0%)^(a)^	(0%)^(a)^	(0%)^(a)^	(60%)^(b)^	(10%)^(a)^	

C: Control group; CE: α-Tocopherol control group; S: Sham group; IRI: Ischemic Reperfusion Injury group; IRIE: α-Tocopherol-treated ischemic reperfusion injury group. Results are expressed as mean ± SD (n = 10).

P value < 0.05 is considered significant.

Groups with different letters are statistically significant (p < 0.001)

## Discussion

Spinal cord injury is usually studied by electrophysiological and histological methods. However, in order to assess the degree of injury and recovery, functional evaluation is crucial [5]. So, the current study was designed on rats with spinal cord reperfusion injury model to evaluate both motor and sensory deficits in addition to other laboratory investigations. Improvement in these deficits was also assessed after α-tocopherol administration.

Overproduction of ROS and free radicals is the possible mechanism operating to modulate the patho-physiological phenomenon associated with nervous system injury [33]. The IRI group of rats in our study showed deterioration of motor function and lost placing stepping reflex and impaired sensory function. This finding indicates that induced vascular ischemia resulted in spinal cord injury with subsequent disturbance of different neurological functions [34].

In the present study, spinal cord homogenate MDA levels, as a marker of lipid peroxidation and AOPP levels, as a marker of protein oxidation, increased significantly in the IRI group compared with the control groups (C, CE, S groups with p < 0.001). The same effects have been reported in spinal cord insults induced by chronic constriction injury of the sciatic nerve in rats [6]. The increased generation of oxidative protein product is correlated with the degree of the produced free radicals [35]. Plasma levels of AOPP are also correlated with MDA and pro-inflammatory cytokines levels, suggesting the role of AOPP as a mediator in oxidative stress [36]. Antioxidant enzymes, as SOD, form the primary defense against reactive oxygen metabolites and have been shown to form an important adaptive response to peroxidative stress [37]. In the present study, SOD increased while CAT enzyme decreased significantly in spinal cord homogenate in IRI rats. Similar results have been shown in neurological injuries in series of researches due to oxidative stress and inhibition of antioxidant defense potential [6]. The reduction of CAT activity may be responsible for a number of deleterious effects in IRI due to the accumulation of H_2_O_2 _[38]. Severe reduction of antioxidants levels in nerve, spinal cord and dorsal root ganglion in rat's neurological injuries indicates high susceptibility of these tissues to oxidative stress [6].

Our study demonstrated that NOx and PGE_2 _levels increased significantly in the spinal cord homogenate among the IRI group of rats. It was suggested that vascular neurological injuries induce iNOS activity with subsequent NO over-production leads to COX-2 activation and PGE_2 _overproduction [39]. Consistent with this, Nogawa et al. [40] found that COX-2 mRNA expression in the brain peaked 12 hours after middle cerebral artery occlusion; at a time when iNOS also reached peak expression as shown by the simultaneous elevation of both NOx and PGE_2 _in the present work. Transient global ischemia in gerbils results in a biphasic increase in COX activity with an early increase in COX-1 activity and a delayed persistent increase in COX-2 activity with subsequent PGE_2 _overproduction [41]. It has been documented that COX-2 and its product PGE_2 _participate in pathogenesis of ischemic injury in the human brain [42].

Our results showed reduction of the lipid peroxidation product "MDA", protein oxidation product "AOPP" and plasma NOx levels in the IRIE group, compared with the control groups. Ziegler et al. stated that α-TOL is one of the most potent biological antioxidants in the body tissues that effectively protects against neuronal oxidative stress damage. It interacts with free radicals and prevents lipid peroxidation [43]. Clinically, α-TOL supplementation led to electrophysiological recovery of sensory conduction and evoked potentials in neurological vascular insults [44]. Experimental studies showed that α-TOL administration in animal's ischemic reperfusion injury not only attenuates the oxidative injury of the muscle cells but also reduces the formation of edema in these cells. This may be due to the anti-inflammatory effect of α-TOL by inhibiting PGE_2 _production [43]. The inhibitory effect of α-TOL on PGE_2 _production in the presence of abundant arachidonic acid, (the substrate for PGE_2_), indicates that α-TOL mediates PGE_2 _inhibition mainly through COX-2 inhibition rather than through substrate release [45]. Wu et al. [46] showed that α-TOL exerts its effect on COX activity and not on the downstream enzyme PGE_2 _isomerase activity. They investigated the effect of α-TOL on another COX product, thromboxane A_2 _in the supernatant from COX activity cultures and they did not find any effect of α-TOL on the isomerase activity. As full activation of COX-2 enzyme requires the presence of sufficient hydroperoxides [10]; α-TOL might attenuate COX-2 activity by scavenging the oxidant hydroperoxide and reducing the formation of peroxynitrite [18]. The fact that α-TOL inhibits COX-2 activity in old mice supports this finding, as formation of lipid peroxides and NO increases in different tissues of the aged animals [18]. Some investigators reported that NO stimulates COX activity via direct stimulation of the enzyme [47]. It is metabolized to peroxynitrite (ONOO) in the presence of superoxide with subsequent induction of COX activity without affecting its expression. α-TOL may acts via other mechanisms than antioxidant activity including cell signaling, interfering with other enzymatic activity, apoptosis and modulating gene expression that may contribute to its neuroprotective action [48].

Administration of α-TOL in IRI rats not only improves the biochemical parameters measured but almost restores the motor function, placing steeping reflex and sensory responses to noxious stimuli up to the normal control levels. This confirms that α-TOL not only improves all aspects of oxidative stress injury and inflammatory responses of the spinal cord in IRI but also its clinical neurological impacts [14]. Interruption of aortic blood flow in IRI may not only induce spinal cord injury, but also induce injury of the heart, gut, and kidney [41]. An additional mechanism that could be responsible for α-TOL long-lasting neuro-protection of neurons against oxidative stress; this was reported by Crouzin et al. who found that, oxidative stress insults activates transient receptor potential vanilloid 1 (TRPV1) channels which one of the members of transient receptor potential (TRP) family in neuronal tissues. Activation of these channels mediate exaggerated Ca^2+ ^influx with subsequent neuronal death. α-TOL pretreatment in oxidative stress conditions down-regulates TRPV1 channels activity [49]. In addition, it has been found that the tocotrienol (TCT), the subfamily of natural vitamin E, possesses powerful neuroprotective activity. Nanomolar TCT significantly attenuates the effects of glutamate on fatty acid levels and on cytosolic phospholipase A_2_. Phospholipase A_2 _activation results in the release of free arachidonic acid within the cell. Through the Src-Lox death pathway, arachidonic acid overproduction results in the formation of 12- hydroperoxyeicosatetraenoic acid which is lethal for cells [50]. While our data showed a neuro-protective effect of α-TOL in reducing the incidence of paraplegia, it may exert further protective effects on other organs. So, more investigations are required to prove or disprove this hypothesis.

In conclusion, i.m. administration of the COX-2 inhibitor α-TOL, at a dose of 600 mg twice weekly for six weeks, significantly prevents the damage caused by spinal cord ischemia in rats. Moreover, α-TOL improves motor and sensory functions and reduces oxidative stress level. Because spinal cord ischemia-induced paraplegia remains a serious complication of IRI, prophylactic α-TOL administration might prove useful in its prevention. However, extrapolating from rats to humans in this entity must be done with caution, and testing with different doses and therapy regimens in some other animal species remains to be completed and thoroughly observed by safety trials in humans before its clinical use.

## Competing interests

The authors declare that they have no competing interests.

## Authors' contributions

MMD participated in the design of the study, performing of the experiments and helping draft the manuscript. OAM participated in the performing the experiment and revised the manuscript and constructed the figures and tables. WNH performed the chemical analysis and participated in revision of the manuscript including figures and tables. All authors have read carefully and approved the final manuscript.
